# Comparative Genomic Analysis of *Streptococcus dysgalactiae subspecies dysgalactiae* Isolated From Bovine Mastitis in China

**DOI:** 10.3389/fmicb.2021.751863

**Published:** 2021-10-22

**Authors:** Siyu Xu, Yang Liu, Jian Gao, Man Zhou, Jingyue Yang, Fumeng He, John P. Kastelic, Zhaoju Deng, Bo Han

**Affiliations:** ^1^Department of Clinical Veterinary Medicine, College of Veterinary Medicine, China Agricultural University, Beijing, China; ^2^Department of Production Animal Health, Faculty of Veterinary Medicine, University of Calgary, Calgary, AB, Canada

**Keywords:** bovine mastitis, *Streptococcus dysgalactiae* subsp. *dysgalactiae*, whole genome sequencing, virulence factor genes, antimicrobial resistance genes

## Abstract

*Streptococcus dysgalactiae* subsp. *dysgalactiae* (SDSD) is one of the most prevalent pathogens causing bovine mastitis worldwide. However, there is a lack of comprehensive information regarding genetic diversity, complete profiles of virulence factors (VFs), and antimicrobial resistance (AMR) genes for SDSD associated with bovine mastitis in China. In this study, a total of 674 milk samples, including samples from 509 clinical and 165 subclinical mastitis cases, were collected from 17 herds in 7 provinces in China from November 2016 to June 2019. All SDSD isolates were included in phylogenetic analysis based on 16S rRNA and multi-locus sequence typing (MLST). In addition, whole genome sequencing was performed on 12 representative SDSD isolates to screen for VFs and AMR genes and to define pan-, core and accessory genomes. The prevalence of SDSD from mastitis milk samples was 7.57% (51/674). According to phylogenetic analysis based on 16S rRNA, 51 SDSD isolates were divided into 4 clusters, whereas based on MLST, 51 SDSD isolates were identified as 11 sequence types, including 6 registered STs and 5 novel STs (ST521, ST523, ST526, ST527, ST529) that belonged to 2 distinct clonal complexes (CCs) and 4 singletons. Based on WGS information, 108 VFs genes in 12 isolates were determined in 11 categories. In addition, 23 AMR genes were identified in 11 categories. Pan-, core and accessory genomes were composed of 2,663, 1,633 and 699 genes, respectively. These results provided a comprehensive profiles of SDSD virulence and resistance genes as well as phylogenetic relationships among mastitis associated SDSD in North China.

## Introduction

*Streptococcus dysgalactiae* is an important bovine mastitis causing pathogen worldwide (Cameron et al., 2016; Gao et al., 2017; de Campos et al., 2021), which could result in economic loss and deteriorated animal welfare (Heikkilä et al., 2018). *Streptococcus dysgalactiae* consists of 2 subspecies: *Streptococcus dysgalactiae* subsp. *equisimilis* (SDSE) and *Streptococcus dysgalactiae* subsp. *dysgalactiae* (SDSD). They can be distinguished based on their hemolytic properties [SDSD (a-hemolytic) and SDSE (b-hemolytic)] on blood agar (Alves-Barroco et al., 2021). SDSD is classically described as an animal pathogen, causing animal diseases, such as bovine mastitis (Zhang et al., 2018), and mostly results in persistent (sub)clinical mastitis (Botrel et al., 2010). Meanwhile, studies indicate that SDSD isolates can also be isolated from human with breast cancer and infect human primary keratinocyte cells *in vitro* (Alves-Barroco et al., 2019b; Koh et al., 2020), indicating the potential to infect human. Therefore, SDSD in cow milk should be considered a threat to public health.

The pathogenicity of *Streptococcus dysgalactiae* attributes to various virulence factors (VFs), which involved in the following categories: adherence, enzyme (including hyaluronidase, mitogenic factor, streptococcal enolase, and streptodornase et al.), immune evasion, immunoreactive antigen, iron uptake, manganese uptake, protease, superantigen, and toxin formation process, based on *Streptococcus* virulence factors database (VFDB). These virulence genes facilitate the process of adherence to epithelial cells and internalization as well as the subsequent dissemination into host cells (O’Halloran et al., 2016; Alves-Barroco et al., 2019a). Abuse of antimicrobials in mastitis treatment contributes the most to the spread of antimicrobial resistance (AMR) in mastitis associated SDSD isolates (McDougall et al., 2014; Cameron et al., 2016; Zhang et al., 2018). Specifically, the emergence of multiple-drug resistant strains can be a major challenge in the treatment of bovine mastitis and is a growing concern for public health (McDougall et al., 2014; El Garch et al., 2020).

Population structure of SDSD remains unknown, although various genotypes of *S. dysgalactiae* have been described (Rato et al., 2013; Santos-Sanches et al., 2015). Genetic characteristics of SDSD associated with bovine mastitis, including virulence factors and AMR genes, have been profiled using PCR-based methods (Tian et al., 2019). However, these methods are insufficient to capture the subtle genomic differences among strains. Whole genome sequencing (WGS) with high resolution could be the preferred method to determine strain diversity and to infer phylogenetic relationships as well as to identify virulence and resistance genes in SDSD isolates with subtle differences. Therefore, the objectives of this study were to (1) estimate the apparent prevalence of SDSD in samples from (sub)clinical mastitis; (2) determine genetic diversity and evolution of SDSD isolates by 16s RNA sequencing and whole genome sequencing analysis; (3) identify VFs and AMR genes in those SDSD isolates.

## Materials and Methods

### Statement of Ethics

This study was conducted in accordance with ethical guidelines and standard biosecurity and institutional safety procedures of China Agricultural University (CAU; Beijing, China). Prior to the start of the study, ethical approval was granted by the Departmental Committee of the College of Veterinary Medicine, CAU.

### Milk Sample Collection and *Streptococcus dysgalactiae* subsp. *dysgalactiae* Identification

A total of 674 milk samples, including 509 clinical mastitis (CM) and 165 subclinical mastitis (SCM) milk samples, were obtained from 17 large dairy farms (each had > 1,000 lactating cows) in 7 provinces (Tianjin = 19 samples; Shanxi = 15 samples; Hebei = 189 samples; Heilongjiang = 87 samples; Inner Mongolia = 95 samples; Shandong = 259 samples; and Shaanxi = 10 samples) in China from November 2016 to June 2019. The CM samples, including changes in the milk (e.g., clots, discoloration, flakes, and wateriness) and either with or without visible abnormalities of the udder and/or systemic symptoms (e.g., red, swollen, firm or painful udder, or fever), were collected from a single quarter with abnormalities. The SCM samples were defined as intramammary infection without clinical symptoms and could be detected by California mastitis test and somatic cell counts (Godden et al., 2017). All milk samples were collected aseptically, stored in an ice box and subsequently transported to Mastitis Reference Laboratory at the College of Veterinary Medicine, CAU, Beijing, China.

Pathogens were identified according to bacteriological culture, colony morphology, gram staining, biochemical tests and 16S rRNA sequencing (NMC, 2017). In short, an aliquot (50 μl) of milk sample was spread on a tryptone soy agar (TSA; Oxoid, Basingstoke, United Kingdom) with 5% defibrinated sheep blood (Land Bridge Technology, Beijing, China) and incubated aerobically at 37°C in a humidified condition for 24 h. Bacterial colony morphology was recorded and samples that yielded ≥ 3 morphologically distinct colonies were considered as contaminated and were excluded from the subsequent analysis. Gram staining and catalase testing were conducted to discriminate *Staphylococci* (gram positive, catalase- positive) and *Streptococci*-*enterococci* group (gram-positive, catalase-negative). Esculin test was used to differentiate esculin-positive cocci and *Streptococci* (esculin-negative). *Streptococci* were identified at subspecies level using 16S rRNA gene sequencing (Beijing Sunbiotech Inc., Beijing, China) and BLAST with sequences deposited in National Center for Biotechnology Information (NCBI). A total of 51 isolates were identified as SDSD and stored in Brain Heart infusion broth (BHI; Aobox, China) containing 25% glycerol and stored at −80 °C for subsequent analysis.

### DNA Extraction

Genomic DNA was extracted with a bacterial genomic DNA extraction kit (CoWin Biosciences, Beijing, China) for each bacterial isolate according to manufacturer’s instructions for Gram-positive bacteria. Additional RNase A (4 μl, 100 mg/ml) was used to exclude RNA. The extracted DNA was quantified with a NanoDrop One spectrophotometer (Thermo Fisher Scientific, Waltham, MA).

### 16S rRNA Phylogenetic Analysis

The 16S rRNA gene sequences of a total of 51 SDSD isolates, along with sequence from the selected reference strain ATCC 43078 (Accession no. NR_115275 REGION: 11.767) from GenBank, were edited and aligned using ClustalW multiple sequence alignment algorithm and then phylogenetic tree was constructed via Maximum Likelihood (Tamura-Nei model) with MEGA-X v10.1.8 (Tamura and Nei, 1993). Confidence values for each branch of the phylogenetic tree was estimated using bootstrapping with 1,000 resamplings. Loci with < 95% site coverage, including fewer than 5% alignment gaps, missing data and ambiguous bases at any positions (partial deletion option) (Kumar et al., 2018), were eliminated. The phylogenetic tree was visualized by the iTOL web server^1^ (Letunic and Bork, 2019).

### Multi-Locus Sequence Typing

MLST was applied to determine sequence types (STs) of the SDSD isolates based on 7 housekeeping genes, namely gki, gt, murI, mutS, recP, xpt, and atoB. Sequence at each locus was assigned with an allele number, and the corresponding combination of the 7 allele numbers for each isolate was submitted to the PubMLST database^2^ to obtain the ST of the isolate. In this study, a clonal complex (CC) was defined as a group of STs in which every ST shared at least 5 of 7 identical allele profiles with at least 1 other ST in the group. The minimum spanning tree (MST) was constructed by the geoBURST algorithm and visualized by the PhyloViz web server^3^ to infer phylogenetic relationships among STs of original isolates.

### Genome Assembly and Annotation

A total of 10 SDSD isolates (of 3 dominant STs) from clinical mastitis cases and 2 from subclinical mastitis cases were randomly selected for whole genome sequencing. The DNA samples with concentration ≥ 40 μg/mL and purity indices A260/280 ≥ 1.8, A260/230 ≥ 2.0 were submitted for whole genome sequencing with an Illumina HiSeq 4000 system (Illumina, San Diego, CA, United States) at the Beijing Genomics Institute (Shenzhen, China). Genomic DNA was sheared randomly to construct 3 read libraries with lengths of 350 bp by a Bioruptor ultrasonicator (Diagenode, Denville, NJ, United States) and physicochemical methods. The paired-end fragment libraries were sequenced according to the Illumina HiSeq 4000 protocol. Raw reads of low quality from paired-end sequencing (those with consecutive bases covered by fewer than 5 reads) were discarded. Subsequently, the reads were assembled using SOAPdenovo Version 1.05.^4^ The total number of reads, sequences, and contigs, genome size, N50 and total length were obtained. Gene prediction was performed on the SDSD genomes assembly by glimmer3^5^ with Hidden Markov models. tRNA, rRNA and sRNAs were identified by tRNAscan-SE, RNAmmer, and the Rfam database, respectively. The tandem repeats annotation was obtained using the Tandem Repeat Finder.^6^ Prophage regions were predicted using the PHAge Search Tool Enhanced Release (PHASTER) web server^7^ (Arndt et al., 2016).

### Whole Genome Analysis

#### Identification of Virulence-Associated Genes and Antimicrobial Resistance Genes

Virulence-associated genes were identified based on the core dataset in VFDB Version 2019–07 (Liu et al., 2019). Antimicrobial resistance genes were identified by comparing the SDSD genomes Comprehensive Antibiotic Research Database (CARD) Version V6. Amino acid sequences of predicted genes were aligned against the proteins in these databases using blastp. A gene was assigned to a virulence or antimicrobial resistance protein by the highest score hit containing a minimum identity of 40%. The *E*-value 5.3E-08 was the highest among those *E*-values. Pseudo genes were excluded from the virulence and AMR genes. Location of the AMR genes were retrieved from MobileElementFinder^8^ and ISFinder^9^ and PHASTER (see text footnote 7).

#### Pan-Genome Analysis

The pan-genome of 12 SDSD isolates was computed with Bacterial Pan Genome Analysis tool (BPGA) Version 1.3, using a USEARCH algorithm to cluster orthologous gene families (Chaudhari et al., 2016). Core genes were defined as the genes that exist in all the genomes, accessory genes were defined as genes present in ≥ 1 genomes but not all the genomes, while unique genes were defined as the genes only found in a single genome, according to BPGA analysis. Functional annotations of core, accessory, and unique genes were obtained after comparing sequences to those present in the clusters of orthologous groups (COGs) of proteins and Kyoto encyclopedia of genes and genomes (KEGG) databases. The phylogenetic tree was constructed based on core genes of 12 SDSD genome sequences, combined with 12 *Streptococci* genome sequences obtained from the National Center for Biotechnology Information (NCBI) (details in Supplementary Table 1) using BPGA v1.3.

### Statistical Analyses

Test for proportions was applied to compare the proportion of the genes fall into the functional categories among core, accessory and unique genome using SPSS 23.0 software (SPSS Inc., Chicago, IL, United States) and significance was considered at *p* < 0.05 in a two-tailed test.

## Results

### Isolates

Detailed information of SDSD isolate is shown in Tables 1, 2. A total of 51 (7.57%) isolates were obtained from 674 milk samples, 49 isolates from 509 CM samples and 2 isolates from 165 SCM samples. The apparent prevalence of SDSD in CM and SCM samples was 49/509 (9.6%) and 2/165 (1.2%), respectively.

**TABLE 1:** *Streptococcus dysgalactiae* subsp. *dysgalactiae* isolates (*n* = 51) recovered from 509 bovine clinical mastitis (CM) and 165 subclinical mastitis (SCM) milk samples collected from 17 large dairy farms in 7 provinces of China.

Province	Farm	Date	Mastitis type	No. samples	No. isolates	Rate (%)
Tianjin	A	2017/09	CM	19	2	10.5
Shanxi	B	2018/01	CM	15	1	6.7
Hebei	C	2018/03	CM	19	8	42.1
Heilongjiang	D	2018/04	CM	26	1	3.8
Hebei	E	2018/04	CM	9	1	11.1
Heilongjiang	F	2018/05	CM	25	1	4.0
Inner Mongolia	G	2018/06	CM	15	2	13.3
Shandong	H	2017/12	CM	9	1	11.1
Hebei	I	2018/06	CM	16	1	6.3
Shaanxi	J	2018/06	CM	10	1	10.0
Heilongjiang	K	2018/09	CM	36	3	8.3
Inner Mongolia	L	2017/06	CM	19	3	15.8
Hebei	M	2017/05	CM	40	2	5.0
Hebei	N	2017/04	CM	25	3	12.0
Inner Mongolia	O	2016/11	CM	61	5	8.2
Shandong	P	2019/03	CM	125	12	9.6
Hebei	Q	2019/06	CM	40	2	5.0
Shandong	P	2019/03	SCM	125	1	0.8
Hebei	Q	2019/06	SCM	40	1	2.5
Total	17	/	/	674	51	7.57

**TABLE 2:** *Streptococcus dysgalactiae* subsp. *dysgalactiae* isolate (*n* = 51) recovered from 509 bovine clinical mastitis (CM) and 165 subclinical mastitis (SCM) milk samples collected from farms in China and their genotypes.

Isolate	Province	Farm	Date	Sample type	Allelic numbers							ST	CC
	
					*gki*	*gtr*	*murI*	*mutS*	*recP*	*xpt*	*atoB*		
SDSD_1	Tianjin	A	2017/09	CM	43	43	35	38	50	69	39	ST460	/
SDSD_2	Tianjin	A	2017/09	CM	43	43	35	38	50	69	39	ST460	/
SDSD_4	Shanxi	B	2018/01	CM	41	43	35	34	50	64	39	ST453	CC1
SDSD_5	Hebei	C	2018/03	CM	40	43	35	34	48	67	40	ST461	CC2
SDSD_6	Hebei	C	2018/03	CM	40	43	35	34	48	67	40	ST461	CC2
SDSD_7	Hebei	C	2018/03	CM	39	43	35	55	48	67	40	ST521	CC2
SDSD_9	Hebei	C	2018/03	CM	40	43	35	38	50	64	39	ST523	CC1
SDSD_10	Hebei	C	2018/03	CM	40	43	35	34	48	67	40	ST461	CC2
SDSD_11	Hebei	C	2018/03	CM	40	43	35	34	48	67	40	ST461	CC2
SDSD_12	Hebei	C	2018/03	CM	40	43	35	34	48	67	40	ST461	CC2
SDSD_13	Hebei	C	2018/03	CM	39	43	35	55	48	67	40	ST521	CC 2
SDSD_15	Heilongjiang	D	2018/04	CM	40	43	35	38	50	64	39	ST523	CC1
SDSD_16	Hebei	E	2018/04	CM	39	43	35	55	48	67	40	ST521	CC2
SDSD_17	Heilongjiang	F	2018/05	CM	41	43	35	34	50	64	39	ST453	CC1
SDSD_19	Inner Mongolia	G	2018/06	CM	41	43	35	55	50	64	39	ST529	CC1
SDSD_20	Inner Mongolia	G	2018/06	CM	41	43	35	34	50	64	39	ST453	CC1
SDSD_26	Shandong	H	2017/12	CM	41	43	35	34	48	67	8	ST527	/
SDSD_27	Hebei	I	2018/06	CM	40	43	35	34	48	67	40	ST461	CC2
SDSD_28	Shaanxi	J	2018/06	CM	41	43	35	36	50	64	39	ST305	CC1
SDSD_29	Heilongjiang	K	2018/09	CM	41	43	35	55	50	67	39	ST454	CC1
SDSD_30	Heilongjiang	K	2018/09	CM	41	43	35	55	50	67	39	ST454	CC1
SDSD_32	Heilongjiang	K	2018/09	CM	41	43	35	55	50	67	39	ST454	CC1
SDSD_34	Inner Mongolia	L	2017/06	CM	39	43	35	55	48	67	40	ST521	CC2
SDSD_35	Inner Mongolia	L	2017/06	CM	39	43	35	55	48	67	40	ST521	CC2
SDSD_36	Hebei	M	2017/05	CM	41	43	35	34	48	67	8	ST527	/
SDSD_37	Hebei	M	2017/05	CM	41	43	35	34	50	64	39	ST453	CC1
SDSD_38	Hebei	N	2017/04	CM	39	43	35	55	48	67	40	ST521	CC2
SDSD_39	Hebei	N	2017/04	CM	39	43	35	55	48	67	40	ST521	CC2
SDSD_40	Hebei	N	2017/04	CM	39	43	35	55	48	67	40	ST521	CC2
SDSD_41	Inner Mongolia	O	2016/11	CM	39	43	35	55	48	67	40	ST521	CC2
SDSD_42	Inner Mongolia	O	2016/11	CM	39	43	35	55	48	67	40	ST521	CC2
SDSD_43	Inner Mongolia	O	2016/11	CM	39	43	35	55	48	67	40	ST521	CC2
SDSD_44	Inner Mongolia	O	2016/11	CM	39	43	35	55	48	67	40	ST521	CC2
SDSD_45	Inner Mongolia	L	2017/06	CM	39	43	35	55	48	67	40	ST521	CC2
SDSD_46	Inner Mongolia	O	2016/11	CM	39	43	35	55	48	67	40	ST521	CC2
SDSD_47	Shandong	P	2019/03	CM	40	43	35	38	50	64	39	ST523	CC1
SDSD_48	Shandong	P	2019/03	CM	41	43	35	34	50	64	39	ST453	CC1
SDSD_49	Shandong	P	2019/03	CM	41	43	35	34	50	64	39	ST453	CC1
SDSD_50	Shandong	P	2019/03	CM	41	43	35	34	50	64	39	ST453	CC1
SDSD_51	Shandong	P	2019/03	CM	39	43	35	33	47	64	39	ST298	/
SDSD_52	Shandong	P	2019/03	CM	41	43	35	34	50	64	39	ST453	CC1
SDSD_53	Shandong	P	2019/03	CM	41	43	35	34	50	64	39	ST453	CC1
SDSD_54	Shandong	P	2019/03	CM	41	43	35	34	50	64	39	ST453	CC1
SDSD_55	Shandong	P	2019/03	CM	40	43	35	38	50	64	39	ST523	CC1
SDSD_56	Shandong	P	2019/03	CM	39	43	35	33	47	64	39	ST298	/
SDSD_57	Shandong	P	2019/03	SCM	40	43	35	55	50	69	40	ST526	/
SDSD_58	Shandong	P	2019/03	CM	40	43	35	38	50	64	39	ST523	CC1
SDSD_59	Shandong	P	2019/03	CM	41	43	35	34	50	64	39	ST453	CC1
SDSD_60	Hebei	Q	2019/06	SCM	39	43	35	55	48	67	40	ST521	CC2
SDSD_62	Hebei	Q	2019/06	CM	41	43	35	55	50	67	39	ST454	CC1
SDSD_63	Hebei	Q	2019/06	CM	41	43	35	55	50	67	39	ST454	CC1

### Phylogenetic Analysis Based on 16S rRNA

**FIGURE 1:**
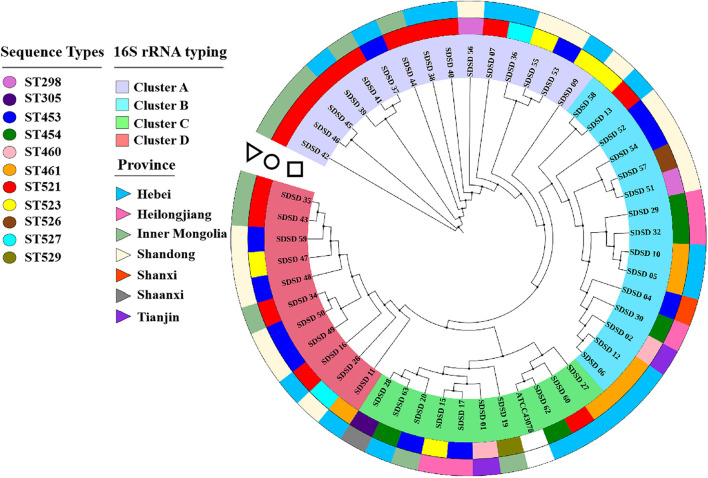
Phylogenetic tree based on 16S rRNA genes (750 bp) of sequence types (STs) and isolation province, with phylogenetic relationships among 51 *Streptococcus dysgalactiae* subsp. *dysgalactiae* (SDSD) isolates, and reference strain ATCC 43078. This tree was constructed with MEGA X and was overlaid with information regarding sequence types (STs) and isolation provinces using the iTOL web server (https://itol.embl.de/). The first ring (circle) indicated the distribution of 51 SDSD isolates into 11 distinct STs (ST298, ST305, ST453, ST454, ST460, ST461, ST521, ST523, ST526, ST527 and ST529). The second ring (triangle) indicated that 51 SDSD isolates were recovered from 7 provinces (Hebei, Heilongjiang, Inner Mongolia, Shandong, Shanxi, Shaanxi, and Tianjin).

The phylogenetic analysis based on 16S rRNA was conducted to determine phylogenetic relationships among the isolates (Figure 1). Four clusters were found: Cluster A consisted of 15 isolates collected from 3 provinces (Hebei, Inner Mongolia and Shandong); Cluster B consisted of 15 isolates from 5 provinces (Hebei, Shandong, Heilongjiang, Shanxi and Tianjin); Cluster C consisted of 10 isolates from 5 provinces (Hebei, Inner Mongolia, Heilongjiang, Shaanxi and Tianjin) and ATCC 43078; and Cluster D consisted of 11 isolates isolated from 3 provinces (Shandong, Inner Mongolia and Hebei). SDSD 62 was the isolate that most closely related to the reference genome ATCC 43078.

### Multi-Locus Sequence Typing and Minimum-Spanning Tree

**FIGURE 2:**
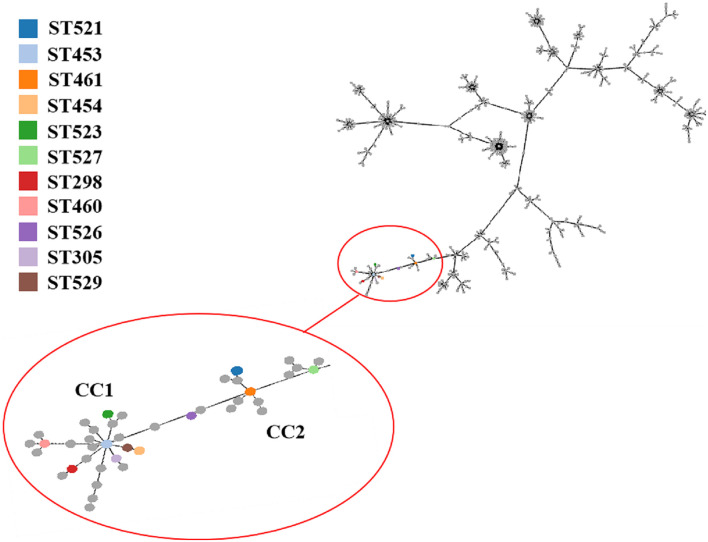
Minimum spanning tree (MST) of MLST for *Streptococcus dysgalactiae* (including *Streptococcus dysgalactiae* subsp. *equisimilis* and subsp. *dysgalactiae*) involving 445 STs in MLST database performed by geoBURST algorithm and visualized by PhyloViz online. The STs identified in this study were in various colors, whereas remaining STs in the MLST database were gray. Cloning complexes (CCs) were defined as groups of similar isolates, each isolate having at least 5 of 7 identical alleles with at least 1 other ST within the group. ST453, ST305, ST523, ST454 and ST529 were grouped into CC1, and ST453 was the main evolutionary starter. ST461, ST521 and ST527 were grouped into CC2, whereas ST460, ST298, and ST526 were considered 3 singletons.

A total of 11 distinct STs were identified in the 51 SDSD isolates from 7 provinces in China (Table 2 and Figure 1), 5 of which were novel, namely ST521, ST523, ST526, ST527, and ST529. Of these STs, ST521 (15 isolates from 7 herds in 2 provinces) was the most predominant one, followed by ST453 (11 isolates from 6 herds in 5 provinces) and ST523 (5 isolates from 3 herds in 3 provinces). The phylogenetic analysis and MST (Figure 2) involving 445 STs were performed. The 11 STs were grouped into 2 CCs and 4 singletons. In CC1, ST453 was the main evolutionary starter and 4 STs were involved, whereas ST461 was the main evolutionary starter in CC2.

### Whole Genome Sequencing, Assembly, and Annotation

In this study, 12 SDSD genomes were sequenced with Illumina HiSeq 4000 system’s protocol. Detailed description on whole genome sequencing, assembly and annotation are shown in Table 3. Seven million reads per isolate and an average genome depth of 59.2 were obtained from the Illumina sequencing. The genome sizes of 12 isolates varied from 2.15 to 2.36 Mb. And the average number of genes identified in all the isolates was 2,042. Furthermore, SDSD47 showed the highest diversity in genes, RNA and prophage regions. Whole genome sequences of the 12 SDSD isolates were deposited in GenBank with the following accession numbers: JAIEZU000000000 (SDSD04), JAIEZX000000000 (SDSD09), JAIFRQ000000000 (SDSD15), JAIEZZ000000000 (SDSD16), JAIEZY000000000 (SDSD17), JAIFAA000000000 (SDSD20), JAIFRR000000000 (SDSD34), JAIFRS000000000 (SDSD37), JAIFRT000000000 (SDSD47); JAIFRU000000000 (SDSD48); JAIFRV000000000 (SDSD57); JAIFRW000000000 (SDSD60).

**TABLE 3:** Sequencing, assembly statistics and annotation of 12 *Streptococcus dysgalactiae* subsp. *dysgalactiae* isolates.

Isolate	Sequencing statistics			Assembly statistics				Annotation					
			
	Total No. reads (×1,000)	Total no. sequences (Mb)	Average read length (bp)	Genome depth	Genome size (Mb)	No. of contigs	N50 (bp)	Total length (Kb)	rRNA	tRNA	sRNA	Tandem repeats	Prophage regions
SDSD04	7,011	1,051	350	58.63	2.27	77	2,055,641	2,035	3	47	38	57	2
SDSD09	7,011	1,051	350	61.96	2.15	81	2,025,205	2,017	3	45	62	31	5
SDSD15	7,011	1,051	350	60.86	2.19	81	2,025,342	2,017	3	45	62	31	5
SDSD16	7,011	1,051	350	58.65	2.27	66	2,070,894	2,025	3	47	41	41	2
SDSD17	7,011	1,051	350	56.42	2.36	85	2,063,783	2,067	3	47	46	38	2
SDSD20	7,011	1,051	350	57.55	2.32	71	2,059,105	2,009	3	47	53	45	1
SDSD34	7,011	1,051	350	60.88	2.19	56	2,029,365	1,964	3	47	44	40	1
SDSD37	7,011	1,051	350	56.44	2.36	74	2,197,553	2,170	3	47	51	57	3
SDSD47	7,011	1,051	350	59.75	2.23	91	2,097,043	2,099	3	45	62	39	6
SDSD48	7,011	1,051	350	59.77	2.23	74	2,042,841	2,038	3	47	47	37	3
SDSD57	7,011	1,051	350	57.61	2.31	78	2,064,798	2,093	3	47	58	47	4
SDSD60	7,011	1,051	350	61.96	2.15	67	2,034,354	1,971	3	47	45	40	1

### Identification of Virulence-Associated Genes

**FIGURE 3:**
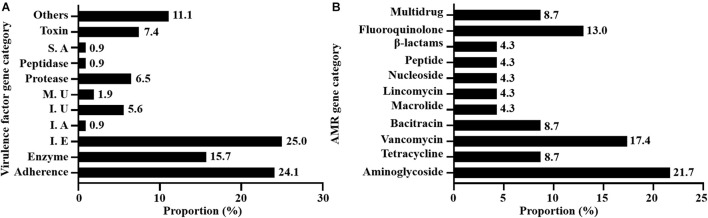
Occurrence of virulence **(A)** and AMR **(B)** genes of 12 SDSD strains isolated from bovine milk samples with clinical and subclinical mastitis in 7 provinces of China. **(A)** There were 11 main virulence categories: adherence, enzyme, immune evasion (I. E), immune reactive antigen (I. A), iron uptake (I. U), manganese uptake (M. U), protease, peptidase, superantigen (S. A), toxin, and others. **(B)** There were 10 AMR categories: aminoglycoside, tetracycline, vancomycin, bacitracin, macrolide, lincomycin, nucleoside, peptide antibiotics, β-lactams, fluoroquinolone, and multidrug.

**FIGURE 4:**
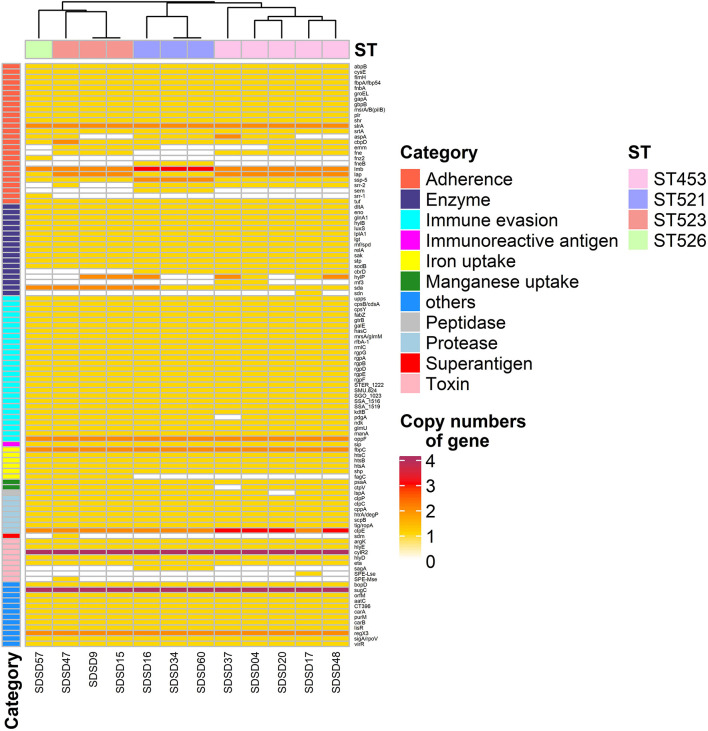
Heatmap of virulence factor gene number distribution in 12 *Streptococcus dysgalactiae* subsp. *dysgalactiae* (SDSD) isolates. A total of 108 virulence-associated genes were determined by Virulence Factors of Database (VFDB) in all 12 SDSD strains, and the copy number, ranging from 0 to 4 were indicated by yellow to red. Eleven main virulence categories were marked with colored bars along the Y-axis. The phylogenetic tree based on the presence of virulence genes of the isolates was presented as a cladogram in the panel along the X-axis.

A total of 108 virulence-associated genes were annotated by VFDB in all 12 SDSD strains, SDSD16 had the highest number of VFs (*n* = 114) and SDSD57 had the least (*n* = 107). These VF genes belonged to 11 main virulence categories: adherence, enzyme, immune evasion, immune reactive antigen, iron, manganese uptake, protease, peptidase, superantigen, toxin-related genes and others. The occurrence and distribution of virulence genes are shown in Figures 3A, 4, respectively.

Among the 26 adherence-related genes, 18 genes (*abpB*, *cysB*, *flmH*, *fbpA*, *fnbA*, *groEL*, *gapA*, *gbpB*, *msrA/B*, *plr*, *shr*, *slrA*, *srtA*, *cbpD*, *lmb*, *lap*, *ssp-5*, and *tuf*) were present in all SDSD isolates. In addition, the number of some genes had ST-specific distributions. For example, isolates of ST521 harbored 3 *lmb* and 2 *ssp-5* gene copies, respectively. Whereas isolates of ST453, ST523, and ST526 contained 2 and 1, respectively.

Regarding enzyme-related genes, *dltA*, *eno*, *glnA1*, *hylB*, *luxS*, *lplA1*, *lgt*, *mf*, *relA*, *sak*, *stp*, *sodB*, and *sda* were present in all SDSD isolates. There were 27 immune evasion-related genes, including 5 capsular genes (*rgpA* to *G*, except *rgpC*) and another 21 immune evasion-related genes were detected in all isolates. Among the 7 protease-related genes present in all STs, *clpE* had uniform distribution: isolates of ST453 had 3 *clpE* copies, whereas isolates of other STs only had 2. Notably, all isolates carried 4 cytolysin regulator gene *cylR2* copies, which belong to the toxin-related genes category.

### Identification of Antimicrobial Resistance Genes

**FIGURE 5:**
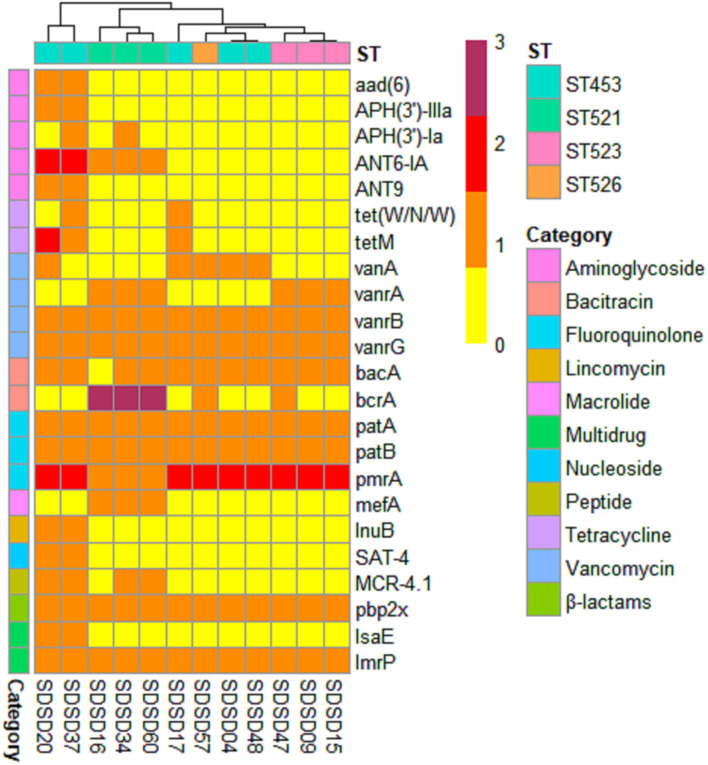
Heatmap of antimicrobial resistance (AMR) gene distribution in 12 *Streptococcus dysgalactiae* subsp. *dysgalactiae* (SDSD) isolates. A total of 23 AMR genes were identified by CARD (Comprehensive Antibiotic Research Database) in all 12 SDSD strains. The copy number, ranging from 0 to 3, was indicated by yellow to red. Ten main AMR categories were marked with colored bars along the Y-axis. The phylogenetic tree based on the presence of AMR genes in the isolates was presented as a cladogram in the panel along the X-axis.

A total of 23 AMR genes in the genomes of 12 SDSD isolates were identified, the occurrence and distribution of AMR genes are presented in Figures 3B, 5, respectively. Based on gene number, 23 AMR genes varied from 0 to 3 copies in the 12 SDSD isolates. Two isolates of ST453, namely SDSD20 and SDSD37, both had the most abundant 21 AMR genes, belonging to 9 distinct classes of antimicrobials (aminoglycoside, tetracycline, vancomycin, bacitracin, fluoroquinolone, lincomycin, nucleoside, peptide, and β-lactamase). Moreover, all 12 isolates harbored 1 *lmrp* gene which encodes a multidrug resistance efflux pump. We found a total of 4 AMR genes (from 4 isolates in ST453 and ST521) were located at mobile genetic elements, most of which were located in insertion sequences [*ANT6-IA*, *SAT-4* and *APH(3′)-IIIA*] and one in transposon [*APH(3′)-Ia*]. Meanwhile, two integrative conjugative elements (ICEs) ICETn6009 were identified in SDSD20 and SDSD37 isolates. However, there was no AMR gene carried by ICETn6009 and prophage regions.

### Pan-Genome Analysis

**FIGURE 6:**
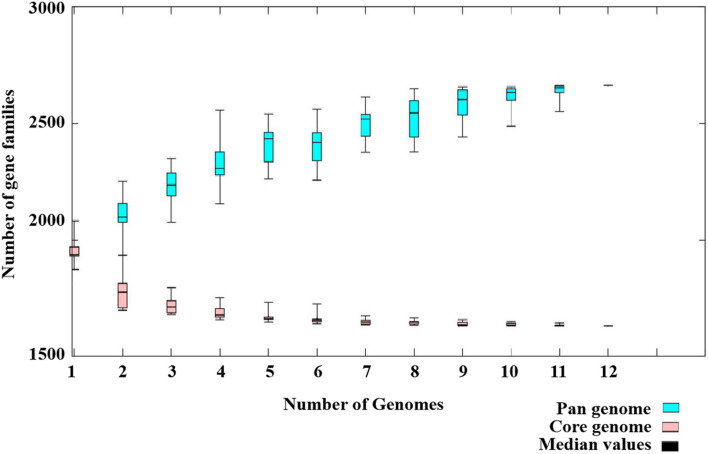
Pan-genome of 12 *Streptococcus dysgalactiae* subsp. *dysgalactiae* (SDSD) isolates. The pan-genome of 12 SDSD tested in this study had 2,663 genes; the size of the genome in the pan-genome continued to increase as the number of strains increased. However, the number of core genomes (shared by 100% of SDSD isolates) was fairly constant at ∼1,600 genes.

**FIGURE 7:**
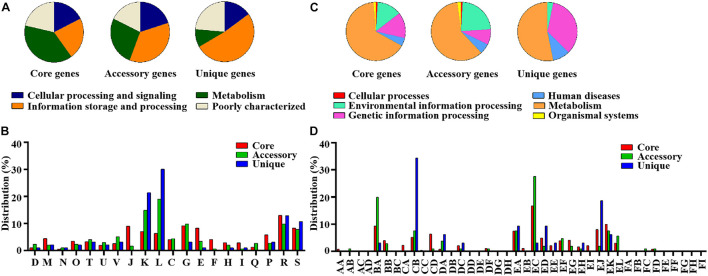
Differential distribution of COG and KEGG functional categories in core, accessory and unique genes: **(A)** Proportion of 4 classes of COG functional categories in core, accessory and unique genes. **(B)** COG functional sub-categories in core, accessory and unique genes. **(C)** Proportion of 6 classes of KEGG functional categories in core, accessory and unique genes. **(D)** KEGG functional sub-categories in core, accessory and unique genes. D, Cell cycle control, cell division, and chromosome partitioning; M, Cell wall/membrane/envelope biogenesis; N, Cell motility; O, Post-translational modification, protein turnover, and chaperones; T, Signal transduction mechanisms; U, Intracellular trafficking, secretion, and vesicular transport; V, Defense mechanisms; J, Translation, ribosomal structure and biogenesis; K, Transcription; L, Replication, recombination and repair; C, Energy production and conversion; G, Carbohydrate transport and metabolism; E, Amino acid transport and metabolism; F, Nucleotide transport and metabolism; H, Coenzyme transport and metabolism; I, Lipid transport and metabolism; Q, Secondary metabolites biosynthesis, transport, and catabolism; P, Inorganic ion transport and metabolism; R, General function prediction only; S, Function unknown. AA, Cell growth and death; AB, Cell motility; AC, Cellular community; AD, Transport and catabolism; BA, Membrane transport; BB, Signal transduction; BC, Signaling molecules and interaction; CA, Folding, sorting and degradation; CB, Replication and repair; CC, Transcription; CD, Translation; DA, Cancers; DB, Cardiovascular diseases; DC, Drug resistance; DD, Endocrine and metabolic diseases; DE, Immune diseases; DF, Infectious diseases; DG, Neurodegenerative diseases; DH, Substance dependence; EA, Amino acid metabolism; EB, Biosynthesis of other secondary metabolites; EC, Carbohydrate metabolism; ED, Energy metabolism; EE, Glycan biosynthesis and metabolism; EF, Lipid metabolism; EG, Metabolism of cofactors and vitamins; EH, Metabolism of other amino acids; EI, Metabolism of terpenoids and polyketides; EJ, Nucleotide metabolism; EK, overview; EL, Xenobiotics biodegradation and metabolism; FA, Circulatory system; FB, Development; FC, Digestive system; FD, Endocrine system; FE, Environmental adaptation; FF, Excretory system; FG, Immune system; FH, Nervous system; FI, Sensory system.

The pan-genome of the 12 SDSD contained 2,663 genes. The core genome (shared by all *S. dysgalactiae* isolates) consisted of 1,633 genes. The accessory genome comprised 699 genes, and the unique genome included 293 genes. The number of core genes was fairly constant at ∼1,600 genes, whereas the size of genes in the pan-genome continued to increase as the number of strains increased (Figure 6). Functional annotation of genes in the pan-genome revealed the distribution of functional categories among 3 pan-genome sets (Figure 7). Among which, metabolism was identified as the most abundant functional category in the core genes. The overall percentage of metabolic functions in the core genes was 38.6%, whereas that in the accessory and unique genes were 26.5 and 9.7%, respectively. Metabolism was almost 1.5 and 4 times more enhanced in the core genes compared to accessory and unique genes (Figure 7A). The functional category of information storage and processing had a higher proportion in unique genes than in core and accessory genes. The functions of transcription and replication, recombination and repair were enhanced in unique genes, whereas the functions of translation, ribosomal structure and biogenesis were enhanced in core genes (Figure 7B). According to blasting with KEGG databases, this distinct distribution was observed (Figure 7C). The functional category of metabolism was the most abundant in three pan-genome sets components, with the overall proportion of 65.7, 60.0 and 53.1% in core, accessory and unique genes, respectively. Notably, the function of carbohydrate metabolism was enhanced in accessory genes rather than in core or unique genes. Meanwhile, the functional category of genetic information processing was significantly enhanced in unique genes than in core or accessory genes. Interestingly, the functions of replication and repair were enhanced in unique genes, whereas, the functions of folding, sorting and degradation, transcription, and translation were enhanced in core genes (Figure 7D).

**FIGURE 8:**
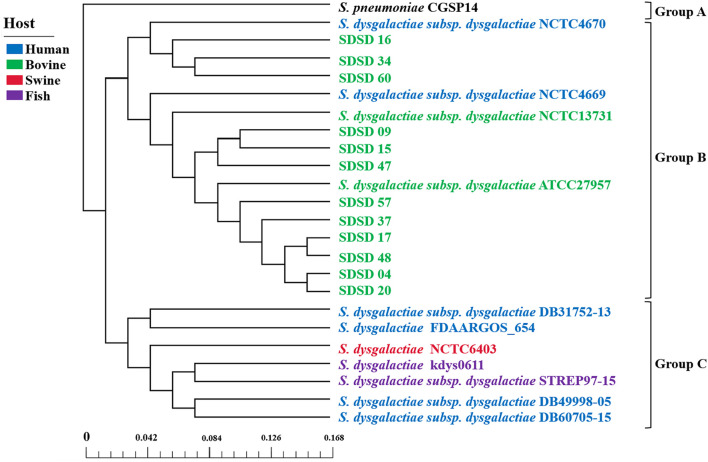
Phylogenetic tree, based on the core genes of 24 *Streptococcus* genomes, including 12 SDSD genomes obtained from the Illumina sequencing, and 12 *Streptococcus* genomes obtained from NCBI. Scale bar indicates the base substitution per site. The phylogenetic tree revealed that the 24 *Streptococcus* genomes were divided into 3 groups. Group A only contained *S. pneumoniae* CGSP14. Group B harbored all SDSD isolates collected from bovine, including 12 SDSD isolates in this study and 2 SDSD genomes (NCTC13731 and ATCC27957) obtained from NCBI. Group C consisted of *S. dysgalactiae* strains obtained from humans (DB31752-13, FDAARGOS_654, DB49998-05 and DB60705-15), fish (kdys0611 and STREP97-15) and swine (NCTC6403).

A phylogenetic tree was constructed based on the core genes of 24 *Streptococci* genomes, including 12 SDSD genomes obtained from the Illumina sequencing and 12 obtained from NCBI (Figure 8). The phylogenetic tree revealed that the 24 *Streptococci* were divided into 3 phylogenetic groups. Group A only contained *S. pneumoniae* CGSP14, and group C included *S. dysgalactiae* strains isolated from human, fish and swine. All SDSD isolates from cattle belonged to group B. Isolates of ST521 were more closely related to SDSD NCTC4670 collected from humans, whereas isolates with ST453, ST523 and ST526 were more closely related to SDSD ATCC27957 and NCTC13731 (both were isolated from dairy cows).

## Discussion

Several studies have described the epidemiology of *Streptococcus dysgalactiae* in dairy herds (Cameron et al., 2016; Horpiencharoen et al., 2019). In addition, studies using PCR-based techniques to identify virulence and AMR genes in bovine *S. dysgalactiae* isolates have been published (Fernandez et al., 2013; Horpiencharoen et al., 2019). However, no in-depth studies have investigated the population structure, profiles of virulence and AMR genes as well as the phylogenetic relationships of bovine mastitis derived SDSD isolates. Therefore, we conducted phylogenetic analysis based on 16S rRNA and MLST of 51 SDSD isolates obtained from 17 dairy herds in 7 provinces of China. In addition, we also conducted WGS of 12 representative SDSD isolates to determine the distribution of virulence and antimicrobial resistance genes. The core genomes were more related to metabolism, and the unique genomes were more relevant to genetic replication and repair according to functional pan-genomes analysis. Consequently, our study suggests that core genes of metabolism can be used as a tool to identify *Streptococcus* species in the microbiome. The phylogenetic tree based on the core genome of 24 *Streptococcus* revealed that human *S. dysgalactiae* isolates have closer genetic relationship with fish and swine *S. dysgalactiae* isolates than bovine SDSD isolates, which is consistent with Alves-Barroco et al. (2021).

The apparent prevalence of SDSD in CM was 9.6%, roughly consistent with the figures in previous studies in China, America, and Europe (Cameron et al., 2016; Soltau et al., 2017; Zhang et al., 2018), but lower than the 28.0% in Australia (Shum et al., 2009). The apparent prevalence of SDSD in SCM samples was 1.2%, lower than other studies (Botrel et al., 2010; Andrea Dieser et al., 2014; Leelahapongsathon et al., 2014).

Understanding the phylogenetic relationships among strains is important to determine the transmission of pathogens. The phylogenetic tree based on 16S rRNA sequences revealed 4 phylogenetic clusters. Cluster A-D contained 15, 15, 10, and 11 isolates derived from 3, 5, 5, and 3 provinces, respectively. Some identical STs (ST521, ST453, ST523, ST454, and ST527) were identified in multiple herds in this study, whereas the other STs were only detected in single herds. That identified STs were in different herds, which is consistent with a previous study (Lundberg et al., 2014). Furthermore, movement of infected animals likely promotes spreading of pathogens between herds (Widgren and Frössling, 2010).

Using the whole genome sequences of 12 representative SDSD genomes, we observed differences in the number of predicted prophage regions in 12 SDSD genomes among different strains and STs. A total of 35 prophage regions were detected in 12 SDSD genomes. The average number of prophages per genome was 2.9-the highest number of prophages was presented in ST453 (5.3) and the lowest was 1.3 in ST521. These values suggest that the number of prophage regions in SDSD could possibly be associated with ST/CC, which is in line with Lichvariková et al. (2020).

Adherence-related genes could facilitate adhesion and biofilm formation, which are important factors in *Streptoccoci* pathogenesis (Widgren and Frössling, 2010). Adhesion is the first step in biofilm formation or invasion into host cells, promoting survival of microorganisms in infected tissues and facilitating development of mastitis (Dahesh et al., 2012; Alves-Barroco et al., 2019a). Eleven adhesion-related genes [*abpB* (Huang et al., 2015), *fbpA* (Bao et al., 2014), *fnBA* (Yeswanth et al., 2017), *shr* (Dahesh et al., 2012), *slrA* (Bober et al., 2011), *cbpD* (Roig-Molina et al., 2020), *fnz2* (Yi et al., 2013), *fneB* (Lannergård et al., 2005), *lmb* (Zhang et al., 2014), seM (Timoney et al., 2010), *srr-1* (Sheen et al., 2011)] encode a number of surface proteins. These surface proteins are identified as important virulence factors that involve bacterial adhesion to the epithelium of the host cell mediated by microbial surface components recognizing adhesive matrix molecules, consequently contributing to host cell attachment and tissue colonization. *Lmb* was the most abundant adherence related gene in the 12 SDSD isolates. In previous studies, the *lmb* gene had a relatively high prevalence in *Streptococcus uberis* isolated from cattle (Fessia et al., 2019), but was less prevalent in bovine-associated *Streptococcus dysgalactiae* and *Streptococcus agalactiae* (Ding et al., 2016; Tian et al., 2019). Three biofilm-related genes (*srtA*, *aspA*, and *emm*) were identified in 12 SDSD genomes. Biofilms are communities of microorganisms attached to a surface and are involved in chronic and recurrent infections in animals and humans (Veerachamy et al., 2014). Compared to planktonic bacteria, those with biofilms are more resistant to antibiotics (Rabin et al., 2015). Therefore, these genes may contribute to the persistent infection induced by SDSD (Kaczorek et al., 2017).

Enzyme (*dltA*, *eno*, *sda*, *hylB*, and *mf*/*spd*), immune evasion (*hasC*, *rgpA* to *G*, except *rgpC*, *oppF* etc.), immune reactive antigen (*sip*), iron and manganese uptake (*shp*, *htsA* to *C*), protease (*htrA*, *clp*), and peptidase (*lspA*) genes were widely distributed among all SDSD. These genes enable *S. dysgalactiae* to colonize (Roy et al., 2019; Diaz-Dinamarca et al., 2020), spread (Florindo et al., 2018) and survive in host tissues (De et al., 2017; Roy et al., 2019) and cause infection (Calonzi et al., 2020). *HylB* was the most abundant enzyme-related gene, and this high prevalence was consistent with previous studies (Ding et al., 2016; Calonzi et al., 2020; Lin et al., 2021). Hyaluronidase encoded by *hylB* promoted intracellular survival of *Streptococci* and expression of pro-inflammatory cytokines, indicating it may have a key role in pathogenesis of *Streptococci* mastitis, including SDSD (Wang et al., 2014).

A total of 23 AMR genes were identified with various copy numbers in 12 SDSD isolates according to the annotation of CARD. According to previous surveys, 5 categories of antimicrobials, including β-lactams (penicillin G, cefalexin, and ceftriaxone), macrolide (erythromycin), aminoglycoside (kanamycin and streptomycin), and tetracyclines, are frequently used in treatment of bovine mastitis on Chinese dairy farms (Shi et al., 2010; Zhang et al., 2018). The emergence of AMR genes are mainly through spontaneous mutations and acquisition of AMR genes from other bacteria (MacLean and Millan, 2019). The high prevalence and breadth diversity of AMR genes, which encode proteins conferring resistance to the 5 categories of antibiotics above, might be attributed to abuse of antimicrobials in Chinese dairy herds. In this study, only one integrative conjugative element *ICETn6009* was detected in SDSD20 and SDSD37, both of which belonged to ST453 and harbored the most abundant AMR genes. *ICETn6009*, a Tn916 based element with Gram-positive *mer* operon and directly linked to the *tet(M)* gene, was identified from two Gram-positive and three Gram-negative genera, including *Streptococcus* (Soge et al., 2008). Meanwhile, 4 AMR genes were located in insertion sequences from 4 isolates in ST453 and ST521 and one in transposon from SDSD 34 in ST521. This may indicate isolates of these two STs were more capable in acquisition of antimicrobial resistance genes from MGEs (Oppegaard et al., 2020).

In general, the average identity values were relatively high for the AMR genes relevant to specific antibiotics: Aminoglycoside [*ANT6-IA*, *APH(3′)-IIIa*, *APH(3′)-Ia*; 100.00%], Peptide (*MCR-4.1*; 100.00%), Lincomycin (*lnuB*; 100.00%), and Nucleoside (*SAT-4*; 99.43%). These high identity values indicate that these genes were highly likely to be AMR genes (Velez et al., 2017). Four antimicrobial resistance mechanisms are involve in the AMR genes identified in 12 SDSD genomes, including antibiotic inactivation [*aad(6)*, *APH(3′)-IIIa*, *APH(3′)-Ia*, *ant6-IA*, *aad9-IB*, *lnuB*, and *SAT4*], antibiotic target alteration (*vanA*, *vanRA*, *vanRB*, *vanRG*, *bacA*, *MCR-4.1*, and *pbp2X*), antibiotic target protection [*tet(W/N/W)*, *tetM*, *mefA* and *IsaE*], and antibiotic efflux (*bcrA*, *patA*, *patB*, and *pmrA*) (Christaki et al., 2020). Aminoglycoside phosphotransferase encoded by *APH(3′)-IIIa* in gram-positive bacteria could phosphorylate the aminoglycoside agents, such as amikacin, neomycin, and kanamycin (Woegerbauer et al., 2014). And *lnuB*, encoding a 3-lincosamide-O-nucleotidyltransferase which could inactivate lincosamides by adenylation in position 3, was reported in *S. agalactiae* (Haenni et al., 2018). Mutation in *pbp2x* could reduce the affinity of the bacteria to β-lactam antibiotics, which has been considered as the major mechanism of *Streptococcus* drug resistance (McDougall et al., 2020; Vannice et al., 2020). *TetM* directly interacts and alters the conformation of nucleotide within 16S rRNA that comprises part of the tetracycline binding site, leading to tetracycline dissociation from the ribosome and prevents rebinding (Dönhöfer et al., 2012). *PatA* and *patB* work as heterodimers to interact together to make a functional drug efflux transporter, conferring efflux-mediated fluoroquinolone resistance in *Streptococcus* (Boncoeur et al., 2012; Baylay et al., 2015). However, the expression of AMR gene is regulated by other components, such as membrane sensor protein (Depardieu et al., 2007). For example, component regulatory systems *bceRS* take part in the regulation of bacitracin resistance in *Streptococcus* (Ouyang et al., 2010). Therefore, AMR phenotypes should be determined to further disclose the relationship between the phenotype and the genotype of SDSD with respect to AMR.

## Conclusion

To the best of our knowledge, this is the first report on characterization of *Streptococcus dysgalactiae* subsp. *dysgalactiae* isolated from mastitis cows in China using whole-genome sequencing. The apparent prevalence of SDSD was estimated at 7.6%. Eleven sequence types (ST298, ST305, ST453, ST454, ST460, ST461, ST521, ST523, ST526, ST527, and ST529) were determined according to MLST. A total of 108 VFs in 11 categories (adherence, enzyme, immune evasion, immune reactive antigen, iron and manganese uptake, protease, peptidase, superantigen, toxin, and others) and 23 AMR genes in 11 categories (aminoglycoside, tetracycline, vancomycin, bacitracin, fluoroquinolone, lincomycin, nucleoside, peptide, macrolide, β-lactams antimicrobials, and multidrug) were identified according to whole genome sequence analysis. These results can be used to study bovine-associated SDSD virulence and resistance genotypes, as well as to better understand the phylogenetic relationships among mastitis-derived SDSD in Northern China.

## Data Availability Statement

The datasets presented in this study can be found in online repositories. The names of the repository/repositories and accession number(s) can be found in the article/Supplementary Material.

## Ethics Statement

This study was conducted in accordance with ethical guidelines and standard biosecurity and institutional safety procedures of China Agricultural University (CAU; Beijing, China). Prior to the start of the study, ethical approval was granted by the Departmental Committee of the College of Veterinary Medicine, CAU.

## Author Contributions

BH conceived and designed the experiment. SX, YL, MZ, JY, FH, JG, and ZD performed the experiments, analyzed the data, and contributed to drafting the manuscript. SX, JG, JK, ZD, and BH wrote, reviewed, and edited the manuscript. All authors approved the final version of the manuscript.

## Conflict of Interest

The authors declare that the research was conducted in the absence of any commercial or financial relationships that could be construed as a potential conflict of interest.

## Publisher’s Note

All claims expressed in this article are solely those of the authors and do not necessarily represent those of their affiliated organizations, or those of the publisher, the editors and the reviewers. Any product that may be evaluated in this article, or claim that may be made by its manufacturer, is not guaranteed or endorsed by the publisher.

## Footnotes

1
https://itol.embl.de/

2
http://pubmlst.org/sdysgalactiae/

3
https://online.phyloviz.net/

4
https://github.com/aquaskyline/SOAPdenovo2

5
http://www.cbcb.umd.edu/software/glimmer/

6
http://tandem.bu.edu/trf/trf.html

7
http://phaster.ca/

8
https://cge.cbs.dtu.dk/services/MobileElementFinder/

9
https://www-is.biotoul.fr/blast.php
