# Supplementary material for: Comparative Genomic Analysis of Streptococcus dysgalactiae subspecies dysgalactiae Isolated From Bovine Mastitis in China
Source: Front Microbiol. 2021 Oct 22;12:751863. doi: 10.3389/fmicb.2021.751863 (PMC8570283; doi:10.3389/fmicb.2021.751863)
Supplement: Supplementary file 1 [file Table_1.docx]

**SUPPLEMENTARY Table S1.** Detail information of 12 *Streptococci* genomes from the National Center for Biotechnology Information (NCBI) used in this study.

| Strain | Species | Source | Accession No. | Assembly Level |
| --- | --- | --- | --- | --- |
| NCTC4669 | *S. dysgalactiae subsp. dysgalactiae* | Human | NZ_LR134094.1 | Complete |
| ATCC27957 | *S. dysgalactiae subsp. dysgalactiae* | Bovine | CM001076.1 | Chromosome |
| STREP97-15 | *S. dysgalactiae subsp. dysgalactiae* | Fish | NZ_CP033166.1 | Chromosome |
| DB31752-13 | *S. dysgalactiae subsp. dysgalactiae* | Human | NZ_CP033164.1 | Chromosome |
| DB49998-05 | *S. dysgalactiae subsp. dysgalactiae* | Human | NZ_CP033163.1 | Chromosome |
| DB60705-15 | *S. dysgalactiae subsp. dysgalactiae* | Human | NZ_CP033165.1 | Chromosome |
| NCTC13731 | *S. dysgalactiae subsp. dysgalactiae* | Bovine | NZ_UHFH01000003.1 | Contig |
| NCTC4670 | *S. dysgalactiae subsp. dysgalactiae* | Human | NZ_UHFG01000004.1 | Contig |
| NCTC6403 | *S. dysgalactiae subsp. equisimilis* | Swine | NZ_LR594046 | Complete |
| kdys0611 | *S. dysgalactiae* | Fish | NZ_AP018726.1 | Complete |
| FDAARGOS_654 | *S. dysgalactiae* | Human | NZ_CP044102.1 | Complete |
| CGSP14 | *S. pneumoniae* | _ | CP001033.1 | Complete |
